# The G-allele of rs10830963 in *MTNR1B* Exerts Stage-Specific Effects Across the Trajectory of Type 2 Diabetes: A Multi-State Analysis

**DOI:** 10.3390/ijms26167855

**Published:** 2025-08-14

**Authors:** Yao Huang, Xiuping Dou, Man He, Yang Su, Hualiang Lin, Yin Yang

**Affiliations:** Department of Epidemiology, School of Public Health, Sun Yat-sen University, Guangzhou 510080, China; huangy863@mail2.sysu.edu.cn (Y.H.); douxp@mail2.sysu.edu.cn (X.D.); heman3@mail2.sysu.edu.cn (M.H.); suyang25@mail2.sysu.edu.cn (Y.S.)

**Keywords:** rs10830963, *MTNR1B*, type 2 diabetes, diabetic microvascular complications, diabetic macrovascular complications, multi-state model

## Abstract

Although the *MTNR1B* single nucleotide polymorphism rs10830963 has been strongly associated with the onset of type 2 diabetes (T2D), its association with the progression and prognosis of T2D has been understudied. We conducted this prospective analysis based on the UK Biobank cohort study. Microvascular complications (MIC) of T2D in this study included diabetic retinopathy, diabetic neuropathy, and diabetic kidney disease. Macrovascular complications (MAC) of T2D included diabetic coronary artery disease, diabetic cerebrovascular disease, and diabetic peripheral vascular disease. The multi-state model was used to analyze the association between the polymorphism of rs10830963 and the trajectory of T2D. The accelerated failure time (AFT) model was used to assess the association between rs10830963 and the onset of T2D and T2D comorbidities. A total of 283,531 middle- and old-age participants were included. During a median follow-up of 13.7 years, 11,947 participants developed T2D, 1556 participants developed MIC, 1797 participants developed MAC, and 618 participants died. In the additive model, the G risk allele of rs10830963 was significantly associated with an increased risk of the transition from T2D-free to T2D (HR = 1.050, 95% CI: 1.020, 1.079) and a decreased risk of the transition from T2D to MIC (HR = 0.918, 95% CI: 0.850, 0.992), particularly from T2D to diabetic retinopathy (HR = 0.882, 95% CI: 0.782, 0.995). Besides, the G risk allele of rs10830963 accelerated the transition from T2D-free to T2D (Time Ratio [TR] = 0.966, 95% CI: 0.947, 0.986) and slowed down the transition from T2D to MIC (TR = 1.067, 95% CI: 1.030, 1.105). The *MTNR1B* single nucleotide polymorphism rs10830963 was associated with an increased risk of T2D and a decreased risk of MIC, particularly diabetic retinopathy among T2D individuals. Our results highlight that rs10830963 might play differential roles in the onset and progression of T2D.

## 1. Introduction

Type 2 diabetes (T2D) is a complex genetic disorder due to the alteration of insulin secretion from pancreatic beta cells [1]. Genome-wide association studies in various populations have consistently demonstrated a significant association of *MTNR1B*-rs10830963 with the risk of T2D [2,3]. Carriers of the rs10830963 G allele exhibit higher *MTNR1B* expression and stronger melatonin signaling in pancreatic islets, with reduced insulin secretion. [4]. *MTNR1B* encodes the G-protein-coupled melatonin receptor 1B (MT2), and melatonin acting through this receptor also exerts effects in extra-pancreatic organs, including the cardiovascular, nervous, and renal systems [5,6,7]. Considering more than half of T2D patients develop diabetic comorbidities later in life, however, whether carrying the G risk allele of rs10830963 modifies the risk of diabetic comorbidities remains unclear. The association between *MTNR1B* rs10830963 and T2D comorbidities has been investigated in recent studies, yet the findings remain inconsistent [8,9]. Specifically, the risk of myocardial infarction increased by 19% among T2D patients per one risk allele increase, with the wild-type CC genotype as the reference [10]. However, no significant association between the minor G allele of rs10830963 and all-cause mortality was observed among T2D patients [11]. Besides, several studies investigated the association between *MTNR1B* rs10830963 and cardiovascular diseases among different populations and also obtained inconsistent results. For example, among the general population, the G risk allele of rs10830963 was found to be associated with an increased risk of stroke [12]. Among night shift workers, a contrary result was observed: the minor G allele of rs10830963 was associated with a decreased risk of stroke [13]. Therefore, further studies are still needed to investigate whether carrying the minor G allele of rs10830963 has an impact on the dynamic progression of T2D, including transitions from T2D-free to T2D, then to diabetic complications, and finally to death.

Using data from the UK Biobank Study, we aimed to comprehensively assess the associations of *MTNR1B* rs10830963 with transitions from baseline to incident T2D, then to diabetic complications, and finally to death using multi-state models, which are an extension of the traditional Cox proportional hazards model [14]. We further investigated the association of *MTNR1B*-rs10830963 and age at onset of outcomes using accelerated failure time models. Understanding the relationships between rs10830963 and the whole course of T2D can help better understand the biological mechanism of melatonin signaling in the incidence and progression of T2D.

## 2. Results

### 2.1. Descriptive Analysis

Baseline characteristics of the study participants according to rs10830963 genotypes are summarized in Table 1.

**Figure 1:**
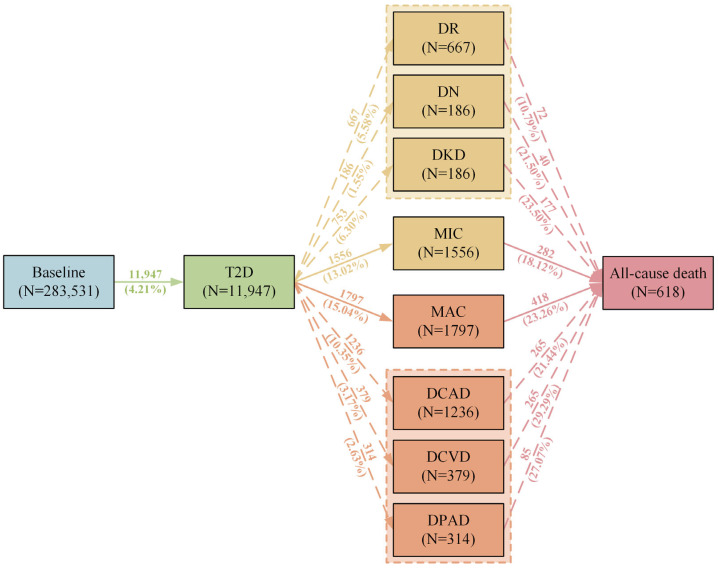
Numbers (percentages) of participants in transitions from baseline to T2D, diabetic comorbidities, and all-cause death. T2D, type 2 diabetes; DR, diabetic retinopathy; DN, diabetic neuropathy; DKD, diabetic kidney disease; MIC, diabetic microvascular complications (including DR, DN, or DKD); DCAD, diabetic coronary artery disease; DCVD, diabetic cerebrovascular disease; DPAD, diabetic peripheral vascular disease; MAC, diabetic macrovascular complications (including DCAD, DCVD, or DPAD).

Among 283,531 participants, the mean age (SD) was 56.06 (8.05) years, 53.45% were women, 56.3% carried two copies of the C allele, 39.7% were heterozygous carriers (i.e., CG or GC), and 7.6% had two copies of the G risk allele. The baseline characteristics were generally consistent among participants with different genotypes, with the exception of age and alcohol intake frequency (both *p* < 0.05). GG carriers were marginally younger than CC carriers (55.96 vs. 56.09 years). For alcohol intake, the proportion of never drinkers was lower in the GG group than the CC group (4.98% vs. 5.47%). During a median follow-up of 13.7 (1.35) years, 11,947 (4.21%) participants transitioned from baseline to T2D, 1556 (13.02%) transitioned from T2D to MIC, 1797 (15.04%) transitioned from T2D to MAC, 282 (18.12%) transitioned from MIC to death, and 418 (23.26%) transitioned from MAC to death (Figure 1).

### 2.2. Associations Between rs10830963 and Trajectory of T2D

**Figure 2:**
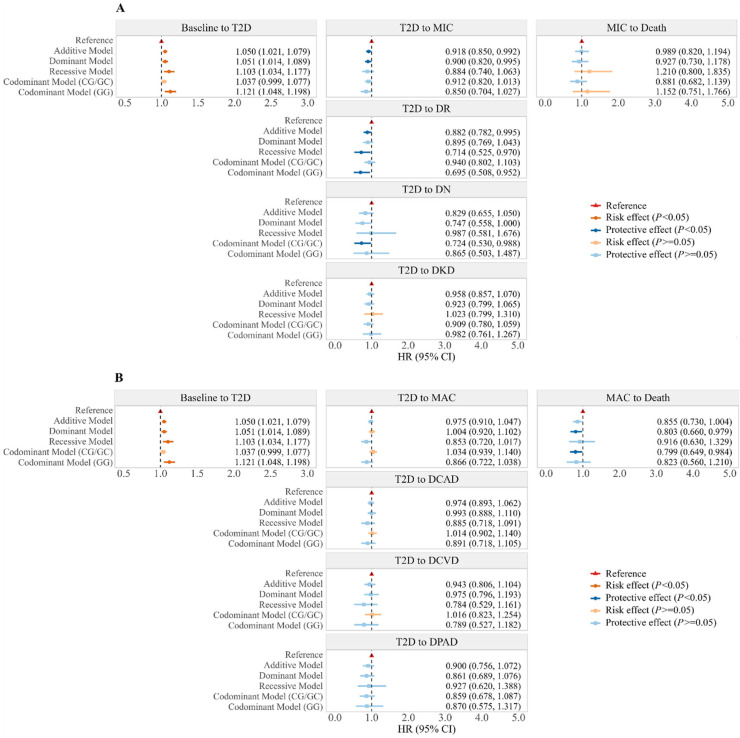
Associations between rs10830963 and transitions from baseline to T2D, T2D comorbidities, and then death. (**A**) Hazard ratios (95% confidence intervals) for transitions from T2D-free to T2D, from T2D to MIC, and from MIC to all-cause death. (**B**) Hazard ratios (95% confidence intervals) for transitions from T2D-free to T2D, from T2D to MAC, and from MAC to all-cause death. HR, hazard ratios; CI, confidence interval; T2D, type 2 diabetes; DR, diabetic retinopathy; DN, diabetic neuropathy; DKD, diabetic kidney disease; MIC, diabetic microvascular complications (including DR, DN, or DKD); DCAD, diabetic coronary artery disease; DCVD, diabetic cerebrovascular disease; DPAD, diabetic peripheral vascular disease; MAC, diabetic macrovascular complications (including DCAD, DCVD, or DPAD).

Results from traditional Cox proportional hazards models showed positive associations between the G risk allele of rs10830963 and the transition from T2D-free to T2D, and negative associations between rs10830963 and the transition from T2D to MIC (particularly DR and DKD) (Table S3). In multi-state models, we observed generally consistent results that rs10830963 played a stage-specific role across the trajectory of T2D (Figure 2 and Table S4).

The G risk allele of rs10830963 was positively associated with the transition from the baseline to T2D, with the risk estimates (HRs) of 1.050 (95% CI: 1.021, 1.079) in the additive model, 1.051 (95% CI: 1.014, 1.089) in the dominant model, and 1.103 (95% CI: 1.034, 1.177) in the recessive model, respectively. However, the G risk allele of rs10830963 was negatively associated with the transition from T2D to MIC. The risk estimates (HRs) were 0.918 (95% CI: 0.850, 0.992) in the additive model, 0.900 (95% CI: 0.820, 0.995) in the dominant model, and 0.884 (95% CI: 0.740, 1.063) in the recessive model, respectively. We further investigated the association between rs10830963 and the transition from T2D to specific MIC, including DR, DN, and DKD. Generally, the G risk allele of rs10830963 was significantly associated with the decreased risk of transition from T2D to DR, with HRs of 0.882 (0.782, 0.995) in the additive model, 0.895 (0.769, 1.043) in the dominant model, and 0.714 (0.525, 0.970) in the recessive model, respectively. However, no significant associations between rs10830963 and the transition from T2D to MAC, MIC to death, and MAC to death were observed.

### 2.3. Associations Between rs10830963 and Onset of T2D and T2D Comorbidities

Using the AFT model, we analyzed the association between rs10830963 and age at onset of T2D and subsequent comorbidities (Table 2).

The findings were generally consistent with the multi-state analyses that the G risk allele of rs10830963 accelerated the transition from baseline to T2D while slowing the progression from T2D to T2D comorbidities. In the additive model, each G allele increase was associated with a 3.4% decrease in the interval from baseline to T2D (TR = 0.966, 95% CI: 0.947–0.986). This directional association remained generally consistent across all genetic models examined. The transition time from T2D to MIC increased by 6.7% per increase in the G allele in the additive model (TR = 1.067, 95% CI: 1.030–1.105), by 8.4% in the dominant model (TR = 1.084, 95% CI: 1.037–1.133), and by 8.7% in the recessive model (TR = 1.087, 95% CI: 1.000–1.182), respectively. For the transitions from T2D to specific MIC, each one G allele increase was associated with a 6.2% prolonged interval from T2D to DR (TR = 1.062, 95% CI: 1.016, 1.110) and a 6.9% prolonged interval from T2D to DKD (TR = 1.069, 95% CI: 1.015, 1.125). However, no significant association between rs10830963 and the onset of MAC was observed in the analysis.

### 2.4. Associations Between rs10830963 and Blood Biochemical Parameters in T2D and Non-T2D Participants

We further analyzed the associations between rs10830963 and blood biochemical parameters related to glycemic regulation among T2D and non-T2D participants (Table S5). The G risk allele of rs10830963 played a totally different role in regulating the levels of HbA1c, SHBG, and IGF-1 among T2D and non-T2D participants. For example, each one G allele increase in rs10830963 was associated with a 0.049 (95% CI: −0.219, 0.121) mmol/mol decrease in HbA1c in T2D participants and a 0.220 (95% CI: 0.202, 0.238) mmol/mol increase in non-T2D participants. Each one G allele increase in rs10830963 was associated with a 0.254 (95% CI: −0.210, 0.717) mmol/L increase in SHBG for T2D participants and a 0.173 (95% CI: −0.280, −0.066) mmol/L decrease for non-T2D participants.

### 2.5. Subgroup and Sensitivity Analyses

We investigated the effect modification of rs1083093 by age, sex, and BMI on the trajectory of T2D (Table S6). The older groups, participants with higher BMI, and females had a higher risk of developing T2D in association with the G risk allele of rs10830963. Besides, those participants were more susceptible to the transition from T2D to MIC associated with the G risk allele of rs10830963 compared with those younger, with lower BMI, and males.

Results from sensitivity analyses remained relatively robust by excluding participants diagnosed with cancer at baseline, excluding participants with T2D that occurred within the first year of follow-up, considering the influence of participants who were diagnosed with T2D and T2D comorbidities on the same day, and additionally adjusting the oral antidiabetic medication use at baseline (Table S7). Similarly, when we calculated the entry date of the prior state using four additional time intervals (0.5 years, 1 year, 3 years, and 5 years) instead of 0.5 days for those participants who entered different stages on the same date, we observed generally consistent results.

## 3. Discussion

In this large-scale cohort study, we examined the association of the single nucleotide polymorphism of *MTNR1B* rs10830963 and the whole course of T2D, from onset to progression to prognosis. We found that rs10830963 played a differential role in multiple transition stages. Specifically, the G risk allele of rs10830963 increased the risk of transition from baseline to T2D and shortened the age at onset of T2D. On the contrary, the G risk allele of rs10830963 decreased the risk of transition from T2D to MIC and extended the age at onset of MIC, particularly DR and DKD.

Single nucleotide polymorphism of *MTNR1B* rs10830963 is known as a driver of T2D in different populations. The adverse effect of the G risk allele on incident T2D observed in the current study was consistent with several prior studies [15,16,17,18,19,20]. On the contrary, the G risk allele of rs10830963 was shown to be associated with a decreased risk of the transition from T2D to MIC. The association between rs10830963 and MIC among T2D participants has not been investigated in previous studies. Besides, we did not find a significant association between rs10830963 and the transition from diabetic complications to death. One previous study investigated the association between rs10830963 and mortality in individuals with T2D and observed null statistical significance, which is generally consistent with our findings [11]. However, these findings obtained from previous studies cannot be compared directly to our estimates due to the distinct analytic strategies and statistical models. Besides, previous studies merely focused on T2D incidence and failed to evaluate the effects of *MTNR1B* rs10830963 on different transition stages of the whole course of T2D, i.e., from T2D-free to T2D, then to T2D comorbidities, and further to death. Furthermore, these studies did not consider the competing risk of death.

The molecular mechanism underlying the observed association between *MTNR1B* rs10830963 and T2D has not been fully clarified [21,22]. Existing evidence showed that the G risk allele of rs10830963 could increase FOXA2-bound enhancer activity in islet- and liver-derived cells, leading to higher expression of *MTNR1B* in human pancreatic islets [23,24]. *MTNR1B* is a G-protein-coupled receptor that mainly acts by interfering with the formation of cAMP through inhibitory G proteins, inhibiting adenylate cyclase, and subsequently inhibiting insulin release [25,26,27]. However, some in vitro and in vivo studies showed that elevated melatonin concentrations could benefit health [28,29]. For example, melatonin exerts pleiotropic vascular protective effects by reducing oxidative and inflammatory stress [30]. Another experimental study demonstrated that melatonin could attenuate diabetic retinopathy by modulating the mesenchymal transition process in retinal vascular endothelial cells [31,32]. This mechanism might partially explain our observed protective effect of the G risk allele of rs10830963 on MIC. Besides, once insulin resistance happens among carriers of the G risk allele in rs10830963, the effect of this mutation on glycemic regulation might disappear. The different associations between rs10830963 and glycemic-related markers among T2D and T2D-free participants might support this hypothesis. We observed that the G risk allele of rs10830963 was associated with increased HbA1c and decreased SHBG levels among T2D-free participants. However, among T2D participants, the G risk allele of rs10830963 was negatively associated with glucose and HbA1c levels, although no statistical significance was observed. Considering the different effects of rs10830963 genotyping on the incidence of T2D and its complications, future studies could use this marker for the risk stratification in T2D and its complications in combination with other parameters.

Our study presents several strengths. The major strength is the use of multi-state models rather than conventional Cox proportional hazards models, which enables us to explore the effect of rs10830963 on different stages of the whole course of T2D while ruling out competing risks. Furthermore, this is the first attempt to investigate the genetic factor on the onset and progression of T2D with the use of an accelerated failure time model. Additionally, the large sample size of the UK Biobank provided substantial power and allowed further investigation of whole transitions of T2D.

Our study also has some limitations. First, although we have adjusted for a series of confounders, there may still be some other residual confounders that we have not considered. For example, information on factors influencing circadian rhythms like light exposure and melatonin supplement intake was lacking, which may have confounded our results. Second, we excluded the individuals with missing data, which could have led to selection bias, although the magnitude of missing data was not large. Third, T2D and T2D comorbidity cases were mainly ascertained by hospital admission records and death registries. The mild T2D cases without hospital admissions may be underestimated. Thus, the association between *MTNR1B* rs10830963 polymorphism and T2D comorbidities may be undervalued; namely, the results may be towards null. Additionally, the small sample size of specific types of T2D comorbidities might result in insufficient power to examine the effect of rs10830963 on T2D complications. Furthermore, to minimize population stratification, we restricted the analyses to participants who self-reported as White British; consequently, the findings are most directly generalizable to individuals of European ancestry. Because allele frequencies and linkage disequilibrium patterns differ across ancestries, the transferability of these associations to other populations remains uncertain. Replication in independent cohorts that include more diverse ancestral backgrounds and larger numbers of specific comorbidity endpoints is required to establish broader applicability.

## 4. Materials and Methods

### 4.1. Study Population

The UK Biobank (UKB) is a prospective cohort study involving more than 500,000 participants, recruited between 2006 and 2010. Socio-demographic, lifestyle, environmental, and medical information of recruited participants was collected at baseline and during follow-up through interviews, questionnaires, and health records. Ethical approval of UK Biobank was obtained from the Northwest Multi-Centre Research Ethics Committee (MREC) and the National Health Service (NHS) National Research Ethics Service on 17 June 2011 (Ref 11/NW/0382) and extended on the 10 May 2016 (Ref 16/NW/0274), and all participants provided written informed consent to participate in the UK Biobank study. This research was conducted under UKB application number 69550.

Initially, 3836 participants were excluded from the analysis if one or more event times were observed beyond the follow-up period to ensure the validity of the time-to-event analysis. Among the remaining 498,575 participants, we excluded participants who self-reported as not being White British (29,435). We also excluded participants with type 1 diabetes (2351), T2D (20,390), microvascular diseases (8707), or macrovascular diseases (21,503) at baseline, as was performed in other cohort studies [33,34]. Additionally, participants with missing data on the *MTNR1B* rs10830963 genotype (11,691) and important covariates (Townsend deprivation index (478), income (1162), education (3371), body mass index (1060), smoking status (1190), alcohol intake (218), physical activity (72,265), diet (25,457), sleep duration (240), and sedentary time (15,526)) were excluded. Finally, 283,531 participants were included in the primary analysis. The detailed selection process was shown in Figure S1. The comparison of baseline characteristics between excluded and included participants was presented in Table S2.

### 4.2. MTNR1B rs10830963 Genotype

The *MTNR1B* rs10830963 genotype was genotyped by the Affymetrix UK Biobank Lung Exome Evaluation (UK BiLEVE) Axiom array or the Applied Biosystems UK Biobank Axiom array. Quality control and imputation were conducted using the Haplotype Reference Consortium, UK10K, and 1000 Genomes phase 3 reference panels. *MTNR1B* rs10830963 was an intronic variant and was on chromosome 11. The minor allele (G allele) frequency of rs10830963 was 27.4% in the current study.

### 4.3. Blood Biochemical Parameter Assessment

Non-fasting venous blood samples were collected from consenting participants at recruitment, and a wide range of biochemical assays were conducted to provide individual data on biomarkers. Blood biomarkers were externally validated with stringent quality control in the UK Biobank; full details on assay performance have been given elsewhere [35]. We included the following 5 blood biochemical parameters related to glycemic regulation in our analysis: hemoglobin A1c (HbA1c, mmol/mol), triglycerides (mmol/L), glucose (mmol/L), sex hormone-binding globulin (SHBG, nmol/L), and insulin-like growth factor 1 (IGF-1, mmol/L). HbA1c was measured using high-performance liquid chromatography analysis on a Bio-Rad VARIANT II Turbo (Bio-Rad Laboratories, Hercules, CA, USA); triglycerides and glucose were analyzed using an enzymatic method on the Beckman Coulter AU5800 platform (Beckman Coulter, Brea, CA, USA); SHBG was analyzed using a chemiluminescent immunoassay two-step sandwich method on the Beckman Coulter DXI 800 platform (Beckman Coulter, Brea, CA, USA); IGF-1 was analyzed using a chemiluminescent Immunoassay one step sandwich method on the DiaSorin Liaison XL platform (DiaSorin S.p.A., Saluggia, Italy).

### 4.4. Ascertainment of Outcomes

T2D was defined by at least one of the following conditions: a medical diagnosis documented in the admission data, self-reported T2D, use of oral hypoglycemic agents, or an HbA1c ≥ 6.5% (48 mmol/mol). Diagnosis of T2D was collected using ICD-9 and ICD-10 codes, with additional exclusions for individuals with a history of type 1 diabetes or other non-gestational diabetes types prior to the onset of T2D. The hospital inpatient records were used to ascertain incident diabetic complications according to ICD-9, ICD-10, and OPCS-4. Microvascular complications (MIC) included diabetic retinopathy (DR), diabetic neuropathy (DN), and diabetic kidney disease (DKD). Macrovascular complications (MAC) included diabetic coronary artery disease (DCAD), diabetic cerebrovascular disease (DCVD), and diabetic peripheral vascular disease (DPAD). The death event was defined as all-cause mortality from death registries in the UK Biobank. The details for diagnoses of outcomes were shown in Table S1.

### 4.5. Covariates

The following covariates were included in the analysis, as they may be related to T2D or T2D comorbidities: age at recruitment, sex, Townsend deprivation index (TDI), annual household income (<GBP [Great British Pound] 18,000, GBP 18,000 to 30,999, GBP 31,000 to 51,999, GBP 52,000 to 100,000 or >GBP 100,000), body mass index (BMI) (underweight (<18.5 kg/m^2^), normal (18.5 to 25 kg/m^2^), overweight (25 to 30 kg/m^2^), or obese (≥30 kg/m^2^)), educational level (any schooling, vocational, college or other), smoking status (never, previous or current), alcohol consumption (never, occasional, moderate, or heavy), sleep duration (7~9 h, <7 h or >9 h), sedentary time (≥4 h, or <4 h), healthy diet (yes or no), physical activity (low vs. moderate vs. high) [36], hypertension status at recruitment, and the first ten principal components of ancestry. The dietary patterns were evaluated according to the recommendations set forth in the Eatwell Guide [37]. These recommendations advocate for the adequate consumption of various foods, including fruits, vegetables, whole grains, fish, shellfish, dairy products, and vegetable oils. Conversely, the recommendations advise limiting the consumption of processed meat, unprocessed meat, and sugar-sweetened beverages. A healthy diet was defined as one that adhered to at least five of the recommendations outlined in the dietary guideline.

### 4.6. Statistical Analysis

Descriptive statistics were used to summarize the sociodemographic characteristics of participants with different alleles of *MTNR1B* rs10830963. Differences between individuals with CC allele, GG allele, or GC/CG allele were compared using chi-squared or Fisher’s exact test for categorical variables and *t*-test for continuous variables.

The associations between *MTNR1B* rs10830963 and outcomes were evaluated using additive (continuous), dominant (GC/CG + GG vs. CC), recessive (GG vs. CC + GC/CG), and codominant models (GC/CG or GG vs. CC). In the initial analysis, we used traditional Cox proportional hazards models to estimate the associations of *MTNR1B* rs10830963 with T2D, MIC, MAC, and all-cause death. We further decomposed these associations and explored the roles of *MTNR1B* rs10830963 in each transitional phase of T2D progression and prognosis, including transitions from T2D-free to T2D, then to diabetic complications, and finally to death by performing multi-state models. All the associations are presented as hazard ratios (HRs) and 95% confidence intervals (CI). By including multiple subsequent or competing events as states of transitions, multi-state models offer a unique advantage in investigating the influence of *MTNR1B* rs10830963 on different stages of T2D progression simultaneously, considering competing risks [38,39]. Several transitions were constructed: (1) Baseline to T2D, (2) T2D to MIC (DR, DN, or DKD), (3) T2D to MAC (DCAD, DCVD, or DPAD), (4) MIC to death, and (5) MAC to death. If diagnoses occurred on the same day, we adjusted the entry date of the previous state to be 0.5 days prior to the entry date of the following state. For example, if a participant was diagnosed simultaneously with T2D and one T2D comorbidity, the T2D event date was recorded as 0.5 days before the date of the T2D comorbidity. Participants diagnosed with multiple complications on the same day were included multiple times in different transitions. Furthermore, the associations between rs10830963 and the age at onset of T2D and T2D comorbidities were analyzed using the Weibull model from the Accelerated Failure Time (AFT) framework, with time ratios (TRs) and 95% CIs reported. AFT models assume that the effect of rs10830963 on the baseline survival curve of T2D or T2D comorbidities is to shrink or stretch this curve [40].

We conducted several sensitivity analyses to evaluate the robustness of the results. To further examine the influence of patients who reached multiple disease states on the same day, we excluded those participants who entered different states on the same date, and we calculated the entering date of the prior state using four additional different time intervals instead of 0.5 days, i.e., 0.5 years, 1 year, 3 years, and 5 years. To exclude the possible influence of delayed diagnosis of an existing T2D at baseline, we reanalyzed the main results after excluding participants with T2D diagnosed within the first year since enrollment. Furthermore, we additionally adjusted for the use of oral antidiabetic medications to exclude the effect of glycemic control. Finally, we repeated the analysis after excluding participants with cancer at baseline.

All analyses were conducted using R software (version 4.3.11). The multi-state models were constructed using the ‘mstate’ package (v 0.3.3). Cox regression models and the AFT models were constructed using the ‘survival’ package (v 3.8-3). Results with a two-sided *p*-value < 0.05 were considered statistically significant.

## 5. Conclusions

Using data from the UK Biobank Study, we found that the G risk allele of rs10830963 was associated with an increased risk of incident T2D and a decreased risk of transition from T2D to microvascular complications, suggesting differential roles of rs10830963 in the incidence and progression of T2D. Future studies should further investigate the potential mechanism under the contrary roles of rs10830963 in the different stages of T2D observed in the current study.

## Figures and Tables

**Table 1:** Baseline characteristics of the UK Biobank population, separated by rs10830963 genotype.

Characteristics	Total (N = 283,531)	rs10830963 Genotype			*P*
		CC (N = 149,392)	CG/GC (N = 112,581)	GG (N = 21,558)	
Mean follow-up, year (SD *)	13.37 (1.82)	13.37 (1.82)	13.37 (1.81)	13.38 (1.83)	0.630
Age, mean (SD)	56.06 (8.05)	56.09 (8.05)	56.04 (8.05)	55.96 (8.06)	0.037
Sex, n (%)					0.253
Male	131,985 (46.55)	69,557 (46.56)	52,284 (46.44)	10,144 (47.05)	
Female	151,546 (53.45)	79,835 (53.44)	60,297 (53.56)	11,414 (52.95)	
Education, n (%)					0.672
Any school degree	113,642 (40.08)	59,892 (40.09)	45,177 (40.12)	8573 (39.77)	
Vocational qualification	18,404 (6.49)	9804 (6.56)	7217 (6.41)	1383 (6.42)	
College education	98,830 (34.86)	51,992 (34.80)	39,252 (34.87)	7586 (35.18)	
Other	52,655 (18.57)	27,704 (18.54)	20,935 (18.60)	4016 (18.63)	
TDI ^‡^, mean (SD)	−1.65 (2.87)	−1.65 (2.87)	−1.65 (2.87)	−1.66 (2.87)	0.792
Income, n (%)					0.390
<GBP ^§^ 18,000	48,081 (16.96)	25,223 (16.88)	19,170 (17.03)	3688 (17.10)	
GBP 18,000 to GBP 30,999	63,420 (22.37)	33,510 (22.43)	25,074 (22.27)	4836 (22.43)	
GBP 31,000 to GBP 51,999	70,661 (24.92)	37,278 (24.95)	27,919 (24.80)	5464 (25.35)	
GBP 52,000 to GBP 100,000	58,000 (20.45)	30,530 (20.44)	23,090 (20.51)	4380 (20.32)	
≥GBP 100,000	15,105 (5.33)	7911 (5.30)	6051 (5.37)	1143 (5.30)	
Unknown	28,264 (9.97)	14,940 (10.00)	11,277 (10.02)	2047 (9.50)	
BMI ^†^, n (%)					0.129
Normal	97,389 (34.35)	51,634 (34.56)	38,468 (34.17)	7287 (33.80)	
Underweight	1352 (0.48)	707 (0.48)	532 (0.47)	113 (0.52)	
Overweight	124,245 (43.82)	65,289 (43.70)	49,501 (43.97)	9455 (43.86)	
Obese	60,545 (21.35)	31,762 (21.26)	24,080 (21.39)	4703 (21.82)	
Alcohol intake frequency, n (%)					0.012
Never	15,285 (5.39)	8163 (5.47)	6049 (5.37)	1073 (4.98)	
Occasional	56,964 (20.09)	29,760 (19.92)	22,829 (20.28)	4375 (20.29)	
Moderate	147,664 (52.08)	78,059 (52.25)	58,406 (51.88)	11,199 (51.95)	
Heavy	63,618 (22.44)	33,410 (22.36)	25,297 (22.47)	4911 (22.78)	
Smoking status, n (%)					0.582
Never	157,382 (55.51)	82,931 (55.51)	62,593 (55.60)	11,858 (55.01)	
Previous	97,887 (34.52)	51,567 (34.52)	38,812 (34.47)	7508 (34.83)	
Current	28,262 (9.97)	14,894 (9.97)	11,176 (9.93)	2192 (10.16)	
Sedentary time, n (%)					0.447
≥4 h	76,136 (26.85)	40,081 (26.83)	30,334 (26.94)	5721 (26.54)	
<4 h	207,395 (73.15)	109,311 (73.17)	82,247 (73.06)	15,837 (73.46)	
Sleep duration, n (%)					0.665
<7 h or >9 h	68,971 (24.33)	36,292 (24.29)	27,473 (24.40)	5206 (24.15)	
7~9 h	214,560 (75.67)	113,100 (75.71)	85,108 (75.60)	16,352 (75.85)	
Healthy diet, n (%)					0.661
Yes	125,199 (44.16)	65,938 (44.14)	49,678 (44.13)	9583 (44.45)	
No	158,332 (55.84)	83,454 (55.86)	62,903 (55.87)	11,975 (55.55)	
Physical activity, n (%)					0.281
Low	50,611 (17.85)	26,747 (17.91)	19,975 (17.74)	3889 (18.04)	
Moderate	116,144 (40.96)	61,328 (41.05)	45,963 (40.83)	8853 (41.07)	
High	116,776 (41.19)	61,317 (41.04)	46,643 (41.43)	8816 (40.89)	
Hypertension, n (%)					0.741
Yes	172,082 (60.69)	58,801 (39.36)	44,155 (39.22)	8493 (39.40)	
No	111,449 (39.31)	90,591 (60.64)	68,426 (60.78)	13,065 (60.60)	

* SD, standard deviation; ^†^ BMI, body mass index; ^‡^ TDI, Townsend deprivation index; ^§^ GBP, Great British Pound.

**Table 2:** Time ratios from the Weibull AFT * model for the transition time from baseline to T2D, T2D comorbidities, and then death.

Transitions	Additive Model (Continuous)	Dominant Model (GC/CG + GG vs. CC)	Recessive Model (GG vs. CC + GC/CG)	Codominant Model	
				GC/CG vs. CC	GG vs. CC
Baseline to T2D	0.966 (0.947, 0.986)	0.965 (0.941, 0.990)	0.932 (0.890, 0.977)	0.974 (0.948, 1.001)	0.922 (0.878, 0.967)
T2D to MIC	1.067 (1.030, 1.105)	1.084 (1.037, 1.133)	1.087 (1.000, 1.182)	1.076 (1.027, 1.127)	1.122 (1.030, 1.222)
T2D to DR	1.062 (1.016, 1.110)	1.055 (0.997, 1.115)	1.176 (1.047, 1.320)	1.031 (0.973, 1.093)	1.191 (1.059, 1.341)
T2D to DN	1.072 (0.974, 1.180)	1.124 (0.997, 1.267)	0.982 (0.793, 1.216)	1.143 (1.006, 1.299)	1.038 (0.833, 1.292)
T2D to DKD	1.069 (1.015, 1.125)	1.099 (1.030, 1.173)	1.045 (0.928, 1.177)	1.102 (1.029, 1.180)	1.088 (0.964, 1.230)
MIC to Death	0.984 (0.949, 1.020)	0.993 (0.948, 1.041)	0.939 (0.866, 1.017)	1.005 (0.956, 1.057)	0.941 (0.866, 1.022)
T2D to MAC	1.030 (0.991, 1.071)	1.027 (0.978, 1.079)	1.078 (0.983, 1.183)	1.016 (0.965, 1.070)	1.086 (0.987, 1.195)
T2D to DCAD	1.021 (0.974, 1.071)	1.017 (0.957, 1.080)	1.064 (0.950, 1.192)	1.007 (0.945, 1.073)	1.067 (0.950, 1.200)
T2D to DCVD	1.064 (0.986, 1.147)	1.061 (0.965, 1.168)	1.156 (0.959, 1.394)	1.040 (0.941, 1.150)	1.176 (0.971, 1.425)
T2D to DPAD	1.044 (0.980, 1.112)	1.088 (1.004, 1.178)	0.958 (0.833, 1.100)	1.109 (1.018, 1.208)	0.999 (0.866, 1.153)
MAC to Death	1.009 (0.976, 1.042)	1.010 (0.970, 1.052)	1.013 (0.937, 1.096)	1.009 (0.966, 1.053)	1.017 (0.939, 1.102)

* AFT, Accelerated Failure Time; the Weibull accelerated failure time model was adjusted for sex, age, Townsend deprivation index, income, education category, body mass index, smoking status, alcohol intake frequency, physical activity, hypertension, healthy diet, sleep duration, sedentary time, and genetic principal components of ancestry (first 10 columns).

## Data Availability

Researchers can use UK Biobank resources to access the data used in this study. UK Biobank Data will be made available on the UK Biobank Consortium website.

## Supplementary Materials

The following supporting information can be downloaded at: https://www.mdpi.com/article/10.3390/ijms26167855/s1.
