# Supplementary material for: The G-allele of rs10830963 in MTNR1B Exerts Stage-Specific Effects Across the Trajectory of Type 2 Diabetes: A Multi-State Analysis
Source: Int J Mol Sci. 2025 Aug 14;26(16):7855. doi: 10.3390/ijms26167855 (PMC12386872; doi:10.3390/ijms26167855)
Supplement: Supplementary file 1 [file ijms-26-07855-s001.zip › ijms-3749634-supplementary.pdf]

Supplemental materials

Supplemental tables

Table S1. Definitions for diagnoses in the UK Biobank study.

Table S2. Baseline characteristics of the included and excluded participants.

Table S3. Associations* of rs10830963 with the incidence of T2D, MIC, MAC, and all-cause death.

Table S4. Associations* between rs10830963 and transitions from baseline to T2D, T2D comorbidities, and then death.

Table S5. Associations between rs10830963 and blood biochemical parameters in T2D and non-T2D participants at baseline.

Table S6. Subgroup analyses of the associations between rs10830963 and transitions from baseline to T2D, T2D comorbidities, and then death.

Table S7. Sensitivity analyses of the associations between rs10830963 and transitions from baseline to T2D, T2D comorbidities, and then death.

Supplemental figure

Figure S1. The flow chart for the inclusion and exclusion of study participants.

Table S1. Definitions for diagnoses in the UK Biobank study.

Diagnosis	Field ID	Codes (If Applicable)
Type 2 diabetes	41270 ICD10	E11, E14
	41271 ICD9	250
	20002 Self–reported	1220, 1223
	20003 Treatment/medication code	Medications for diabetes
	30750 HbA1c	≥48.0 mmol/mol
Diabetic Retinopathy	41270 ICD10	E11.3, E14.3, H33.X, H35.X, H36.0, H43.0, H43.1, H43.8, H54.0, H54.1
	41271 ICD9	2503.0, 2503.2, 3620
	41272 OPCS4	C791, C792, C811, C812, C818, C819, C85
Diabetic Neuropathy	41270 ICD10	E11.4, E14.4, G59.0, G62.9, G63.2, G99.0
	41271 ICD9	2505.0, 2505.2, 3572, 3558
	41272 OPCS4	A733
Diabetic Kidney Disease	41270 ICD10	E11.2, E14.2, N08.3, N18.X, N19, I12.0, I13.X, Z99.2
	41271 ICD9	2504.0, 2504.2, 585, 4039
	41272 OPCS4	M01, X40
Diabetic Coronary Artery Disease	41270 ICD10	I20.0, I20.1, I20.8, I21, I22, I23, I24, I25, I46
	41271 ICD9	410, 411, 412, 413, 414
	41272 OPCS4	K40, K41, K42, K43, K44, K45, K46, K47, K48, K49, K50, K75
Diabetic Cerebrovascular Disease	41270 ICD10	I60, I61, I62, I63, I64, G45.9
	41271 ICD9	430, 431, 432, 433, 434, 435, 438
	41272 OPCS4	L34, L35.1, L35.3, L35.4, L35.8, L35.9
Diabetic Peripheral Artery Disease	41270 ICD10	E11.5, E14.5, I70, I70.2, I70.8, I70.9, I71, I72, I73.8, I73.9, M14.2
	41271 ICD9	440, 441, 442, 4439
	41272 OPCS4	L19, L261, L262, L265, L266, L267, L27, L28, L431, L435, L521, L522, L541, L544, L601, L602, L603, L604, L631, L635, X092, X093, X094, X095, X098, X099, X101, X104, X108, X109, X111, X112, X118, X119

Table S2. Baseline characteristics of the included and excluded participants.

Characteristics	Overall participants (N=502,411)	Excluded participants (N=218,880)	Included participants (N=283,531)	*P*
Age, mean (SD)	56.53 (8.10)	57.13 (8.11)	56.06 (8.05)	<0.001
Sex, n (%)				<0.001
Male	229084 (45.60)	97099 (44.36)	131985 (46.55)	
Female	273327 (54.40)	121781 (55.64)	151546 (53.45)	
Education, n (%)				<0.001
Any school degree	187370 (38.06)	73728 (35.32)	113642 (40.08)	
Vocational qualification	32724 (6.65)	14320 (6.86)	18404 (6.49)	
College education	161130 (32.74)	62300 (29.84)	98830 (34.86)	
Other	111058 (22.55)	58403 (27.98)	52655 (18.57)	
Missing	10129	10129	-	
TDI, mean (SD)	-1.29 (3.09)	-0.83 (3.30)	-1.65 (2.87)	<0.001
Income, n (%)				<0.001
< £ 18 000	97182 (19.58)	49101 (23.07)	48081 (16.96)	
£ 18 000 to £ 30 999	108157 (21.79)	44737 (21.02)	63420 (22.37)	
£ 31 000 to £ 51 999	110753 (22.31)	40092 (18.83)	70661 (24.92)	
£ 52 000 to £ 100 000	86250 (17.37)	28250 (13.27)	58000 (20.45)	
≥ £ 100 000	22927 (4.62)	7822 (3.67)	15105 (5.33)	
Unknown	71131 (14.33)	42867 (20.14)	28264 (9.97)	
Missing	6011	6011	-	
BMI, n (%)				<0.001
Normal	162372 (32.52)	64983 (30.12)	97389 (34.35)	
Underweight	2626 (0.53)	1274 (0.59)	1352 (0.48)	
Overweight	212079 (42.47)	87834 (40.70)	124245 (43.82)	
Obese	122230 (24.48)	61685 (28.59)	60545 (21.35)	
Missing	3104	3104	-	
Alcohol intake frequency, n (%)				<0.001
Never	40627 (8.11)	25342 (11.66)	15285 (5.39)	
Occasional	113837 (22.73)	56873 (26.16)	56964 (20.09)	
Moderate	244694 (48.85)	97030 (44.64)	147664 (52.08)	
Heavy	101753 (20.31)	38135 (17.54)	63618 (22.44)	
Missing	1500	1500	-	
Smoking status, n (%)				<0.001
Never	273476 (54.76)	116094 (53.76)	157382 (55.51)	
Previous	173025 (34.64)	75138 (34.80)	97887 (34.52)	
Current	52962 (10.60)	24700 (11.44)	28262 (9.97)	
Missing	2948	2948	-	
Sedentary time, n (%)				<0.001
≥4h	145492 (30.72)	69356 (36.48)	76136 (26.85)	
<4h	328141 (69.28)	120746 (63.52)	207395 (73.15)	
Missing	28778	28778	-	

Sleep duration, n (%)				<0.001
<7h or >9h	132470 (26.59)	63499 (29.58)	68971 (24.33)	
7~9h	365727 (73.41)	151167 (70.42)	214560 (75.67)	
Missing	4214	4214	-	
Healthy diet, n (%)				<0.001
Yes	196376 (44.45)	71177 (44.97)	125199 (44.16)	
No	306035 (55.55)	87084 (55.03)	158332 (55.84)	
Missing	60619	60619	-	
Physical activity, n (%)				<0.001
Low	76194 (18.94)	25583 (21.54)	50611 (17.85)	
Moderate	164000 (40.86)	47856 (40.29)	116144 (40.96)	
High	162112 (40.30)	45336 (38.17)	116776 (41.19)	
Missing	100105	10015	-	
Hypertension, n (%)				<0.001
Yes	216811 (43.15)	105362 (48.14)	111449 (39.31)	
No	285600 (56.85)	113518 (51.86)	172082 (60.69)	

Abbreviations: BMI, body mass index; SD, standard deviation; TDI, Townsend deprivation index.

Table S3. Associations* of rs10830963 with the incidence of T2D, MIC, MAC, and all-cause death.

Outcomes	Additive Model (Continuous)	Dominant Model (GC/CG + GG vs CC)	Recessive Model (GG vs CC + GC/CG)	Codominant Model	
				GC/CG vs CC	GG vs CC
T2D	1.050 (1.021, 1.079)	1.051 (1.014, 1.089)	1.103 (1.034, 1.177)	1.036 (0.997, 1.075)	1.122 (1.049, 1.199)
MIC	0.887 (0.833, 0.945)	0.861 (0.795, 0.933)	0.856 (0.736, 0.995)	0.873 (0.802, 0.949)	0.804 (0.688, 0.938)
DR	0.874 (0.792, 0.964)	0.886 (0.783, 1.003)	0.697 (0.540, 0.900)	0.934 (0.821, 1.062)	0.671 (0.516, 0.873)
DN	0.874 (0.727, 1.049)	0.798 (0.635, 1.004)	1.035 (0.686, 1.560)	0.771 (0.604, 0.985)	0.910 (0.597, 1.388)
DKD	0.896 (0.824, 0.973)	0.855 (0.769, 0.949)	0.929 (0.766, 1.126)	0.851 (0.762, 0.951)	0.862 (0.707, 1.051)
MAC	0.959 (0.909, 1.011)	0.963 (0.899, 1.031)	0.900 (0.791, 1.023)	0.979 (0.911, 1.051)	0.888 (0.778, 1.013)
DCAD	0.972 (0.913, 1.034)	0.978 (0.903, 1.058)	0.921 (0.794, 1.068)	0.991 (0.912, 1.077)	0.911 (0.782, 1.062)
DCVD	0.910 (0.812, 1.020)	0.913 (0.791, 1.055)	0.803 (0.605, 1.065)	0.942 (0.810, 1.095)	0.784 (0.586, 1.048)
DPAD	0.923 (0.822, 1.036)	0.856 (0.739, 0.991)	1.082 (0.838, 1.397)	0.827 (0.707, 0.968)	1.017 (0.781, 1.323)
All-cause death	0.987 (0.965, 1.009)	0.988 (0.960, 1.016)	0.970 (0.919, 1.023)	0.988 (0.959, 1.017)	0.960 (0.909, 1.015)

Abbreviations: T2D, type 2 diabetes; DR, diabetic retinopathy; DN, diabetic neuropathy; DKD, diabetic kidney disease; MIC, diabetic microvascular complications (including DR, DR or DKD); DCAD, diabetic coronary artery disease; DCVD, diabetic cerebrovascular disease; DPAD, diabetic peripheral vascular disease; MAC, diabetic macrovascular complications (including DCAD, DCVD, or DPAD).

*Cox proportional hazards model was adjusted for sex, age, Townsend deprivation index, income, education category, body mass index, smoking status, alcohol intake frequency, physical activity, hypertension, healthy diet, sleep duration, sedentary time, genetic principal components of ancestry (first 10 columns).

Table S4. Associations* between rs10830963 and transitions from baseline to T2D, T2D comorbidities, and then death.

Transitions	Additive Model (Continuous)	Dominant Model (GC/CG+ GG vs CC)	Recessive Model (GG vs CC + GC/CG)	Codominant Model	
				GC/CG vs CC	GG vs CC
Baseline to T2D	1.050 (1.021, 1.079)	1.051 (1.014, 1.089)	1.103 (1.034, 1.177)	1.037 (0.999, 1.077)	1.121 (1.048, 1.198)
T2D to MIC	0.918 (0.850, 0.992)	0.900 (0.820, 0.995)	0.884 (0.740, 1.063)	0.912 (0.820, 1.013)	0.850 (0.704, 1.027)
T2D to DR	0.882 (0.782, 0.995)	0.895 (0.769, 1.043)	0.714 (0.525, 0.970)	0.940 (0.802, 1.103)	0.695 (0.508, 0.952)
T2D to DN	0.829 (0.655, 1.050)	0.747 (0.558, 1.000)	0.987 (0.581, 1.676)	0.724 (0.530, 0.988)	0.865 (0.503, 1.487)
T2D to DKD	0.958 (0.857, 1.070)	0.923 (0.799, 1.065)	1.023 (0.799, 1.310)	0.909 (0.780, 1.059)	0.982 (0.761, 1.267)
MIC to Death	0.989 (0.820, 1.194)	0.927 (0.730, 1.178)	1.210 (0.800, 1.835)	0.881 (0.682, 1.139)	1.152 (0.751, 1.766)
T2D to MAC	0.975 (0.910, 1.047)	1.004 (0.920, 1.102)	0.853 (0.720, 1.017)	1.034 (0.939, 1.140)	0.866 (0.722, 1.038)
T2D to DCAD	0.974 (0.893, 1.062)	0.993 (0.888, 1.110)	0.885 (0.718, 1.091)	1.014 (0.902, 1.140)	0.891 (0.718, 1.105)
T2D to DCVD	0.943 (0.806, 1.104)	0.975 (0.796, 1.193)	0.784 (0.529, 1.161)	1.016 (0.823, 1.254)	0.789 (0.527, 1.182)
T2D to DPAD	0.900 (0.756, 1.072)	0.861 (0.689, 1.076)	0.927 (0.620, 1.388)	0.859 (0.678, 1.087)	0.870 (0.575, 1.317)
MAC to Death	0.855 (0.730, 1.004)	0.803 (0.660, 0.979)	0.916 (0.630, 1.329)	0.799 (0.649, 0.984)	0.823 (0.560, 1.210)

Abbreviations: T2D, type 2 diabetes; DR, diabetic retinopathy; DN, diabetic neuropathy; DKD, diabetic kidney disease; MIC, diabetic microvascular complications (including DR, DR or DKD); DCAD, diabetic coronary artery disease; DCVD, diabetic cerebrovascular disease; DPAD, diabetic peripheral vascular disease; MAC, diabetic macrovascular complications (including DCAD, DCVD, or DPAD).

*Multi-state model was adjusted for sex, age, Townsend deprivation index, income, education category, body mass index, smoking status, alcohol intake frequency, physical activity, hypertension, healthy diet, sleep duration, sedentary time, genetic principal components of ancestry (first 10 columns).

Table S5. Associations between rs10830963 and blood biochemical parameters in T2D and non-T2D participants at baseline.

	T2D		Non-T2D	
	β (95% CI)	*P*	β (95% CI)	*P*
Glucose (mmol/L)	−0.013 (−0.040, 0.013)	0.320	−0.006 (−0.009, −0.004)	<0.001
HbA1c (mmol/mol)	−0.049 (−0.219, 0.121)	0.571	0.220 (0.202, 0.238)	<0.001
Triglycerides (mmol/L)	0.010 (−0.011, 0.031)	0.371	0.005 (0.001, 0.008)	0.023
SHBG (nmol/L)	0.254 (−0.210, 0.717)	0.283	−0.173 (−0.280, −0.066)	0.002
IGF-1 (mmol/L)	0.076 (−0.075, 0.227)	0.321	−0.010 (−0.038, 0.018)	0.475

Abbreviations: T2D, type 2 diabetes; HbA1c, hemoglobin A1c; SHBG, sex hormone-binding globulin: IGF-1, insulin-like growth factor 1; CI, confidence interval.

Table S6. Subgroup analyses of the associations between rs10830963 and transitions from baseline to T2D, T2D comorbidities, and then death.

	Transitions	Additive Model	Dominant Model	Recessive Model	Codominant Model (CG/GC)	Codominant Model (GG)
Age*						
<58	Baseline to T2D	1.029 (0.982, 1.079)	1.031 (0.973, 1.092)	1.037 (0.933, 1.153)	1.028 (0.968, 1.093)	1.042 (0.934, 1.163)
	T2D to MIC	0.932 (0.776, 1.120)	0.862 (0.688, 1.080)	1.157 (0.747, 1.793)	0.828 (0.651, 1.053)	1.068 (0.690, 1.651)
	T2D to DR	0.856 (0.648, 1.131)	0.840 (0.599, 1.178)	0.898 (0.455, 1.773)	0.840 (0.589, 1.196)	0.844 (0.424, 1.680)
	T2D to DN	0.903 (0.610, 1.336)	0.777 (0.468, 1.288)	1.264 (0.532, 3.004)	0.728 (0.425, 1.246)	1.110 (0.430, 2.863)
	T2D to DKD	1.005 (0.767, 1.318)	0.951 (0.676, 1.338)	1.264 (0.694, 2.303)	0.911 (0.635, 1.307)	1.191 (0.636, 2.231)
	MIC to Death	1.290 (0.766, 2.171)	1.396 (0.675, 2.887)	0.489 (0.104, 2.298)	1.106 (0.485, 2.524)	1.213 (0.367, 4.006)
	T2D to MAC	1.099 (0.965, 1.253)	1.104 (0.936, 1.302)	1.201 (0.888, 1.624)	1.081 (0.910, 1.285)	1.242 (0.908, 1.697)
	T2D to DCAD	1.049 (0.900, 1.223)	1.022 (0.843, 1.240)	1.189 (0.839, 1.686)	1.000 (0.816, 1.224)	1.159 (0.805, 1.668)
	T2D to DCVD	1.262 (0.929, 1.715)	1.476 (0.982, 2.218)	0.708 (0.283, 1.773)	1.563 (1.032, 2.367)	0.948 (0.368, 2.442)
	T2D to DPAD	1.297 (0.915, 1.838)	1.127 (0.705, 1.802)	2.184 (1.129, 4.223)	0.973 (0.585, 1.619)	2.064 (1.004, 4.245)
	MAC to Death	0.546 (0.363, 0.823)	0.458 (0.281, 0.748)	0.575 (0.210, 1.577)	0.471 (0.280, 0.791)	0.408 (0.145, 1.147)
>=58	Baseline to T2D	1.061 (1.024, 1.100)	1.058 (1.011, 1.108)	1.143 (1.053, 1.241)	1.039 (0.990, 1.090)	1.162 (1.067, 1.264)
	T2D to MIC	0.909 (0.833, 0.993)	0.900 (0.804, 1.008)	0.841 (0.683, 1.036)	0.921 (0.817, 1.037)	0.812 (0.656, 1.006)
	T2D to DR	0.883 (0.772, 1.010)	0.907 (0.764, 1.077)	0.686 (0.487, 0.968)	0.961 (0.804, 1.149)	0.675 (0.474, 0.960)
	T2D to DN	0.775 (0.571, 1.051)	0.684 (0.469, 0.998)	0.917 (0.462, 1.819)	0.665 (0.444, 0.996)	0.779 (0.387, 1.568)
	T2D to DKD	0.946 (0.837, 1.069)	0.910 (0.777, 1.067)	0.999 (0.761, 1.311)	0.900 (0.760, 1.065)	0.955 (0.721, 1.264)
	MIC to Death	1.010 (0.823, 1.240)	0.923 (0.710, 1.198)	1.359 (0.876, 2.109)	0.854 (0.645, 1.132)	1.276 (0.812, 2.006)
	T2D to MAC	0.923 (0.846, 1.007)	0.958 (0.856, 1.072)	0.737 (0.592, 0.916)	1.010 (0.898, 1.137)	0.740 (0.592, 0.926)
	T2D to DCAD	0.939 (0.844, 1.044)	0.977 (0.851, 1.121)	0.762 (0.586, 0.990)	1.026 (0.888, 1.185)	0.771 (0.588, 1.009)
	T2D to DCVD	0.847 (0.702, 1.022)	0.806 (0.635, 1.024)	0.825 (0.532, 1.279)	0.818 (0.635, 1.053)	0.757 (0.483, 1.187)
	T2D to DPAD	0.786 (0.639, 0.965)	0.768 (0.593, 0.994)	0.632 (0.373, 1.069)	0.813 (0.620, 1.065)	0.579 (0.339, 0.990)
	MAC to Death	0.934 (0.781, 1.118)	0.884 (0.708, 1.104)	1.065 (0.707, 1.604)	0.866 (0.687, 1.092)	0.994 (0.651, 1.519)

Sex[†]						
Female	Baseline to T2D	1.068 (1.023, 1.115)	1.071 (1.013, 1.132)	1.151 (1.043, 1.270)	1.053 (0.993, 1.116)	1.165 (1.052, 1.290)
	T2D to MIC	0.845 (0.751, 0.951)	0.814 (0.700, 0.945)	0.787 (0.594, 1.044)	0.833 (0.711, 0.977)	0.731 (0.548, 0.975)
	T2D to DR	0.875 (0.736, 1.039)	0.869 (0.697, 1.082)	0.738 (0.481, 1.131)	0.902 (0.716, 1.136)	0.728 (0.472, 1.124)
	T2D to DN	0.776 (0.516, 1.167)	0.774 (0.471, 1.271)	0.529 (0.163, 1.715)	0.826 (0.494, 1.380)	0.519 (0.160, 1.676)
	T2D to DKD	0.856 (0.722, 1.014)	0.799 (0.643, 0.993)	0.877 (0.598, 1.288)	0.796 (0.631, 1.004)	0.812 (0.548, 1.203)
	MIC to Death	1.139 (0.840, 1.543)	1.166 (0.784, 1.734)	1.237 (0.610, 2.507)	1.138 (0.746, 1.736)	1.297 (0.628, 2.679)
	T2D to MAC	0.953 (0.838, 1.085)	1.119 (0.918, 1.364)	0.908 (0.672, 1.227)	0.965 (0.809, 1.150)	0.894 (0.655, 1.219)
	T2D to DCAD	0.980 (0.839, 1.144)	0.962 (0.786, 1.176)	1.015 (0.715, 1.440)	0.955 (0.771, 1.181)	0.993 (0.691, 1.427)
	T2D to DCVD	0.914 (0.703, 1.189)	0.932 (0.665, 1.306)	0.748 (0.391, 1.432)	0.972 (0.683, 1.385)	0.760 (0.392, 1.473)
	T2D to DPAD	0.906 (0.625, 1.313)	0.863 (0.538, 1.385)	0.944 (0.405, 2.201)	0.857 (0.519, 1.415)	0.891 (0.373, 2.125)
	MAC to Death	0.841 (0.629, 1.123)	0.849 (0.584, 1.233)	0.637 (0.305, 1.331)	0.914 (0.615, 1.360)	0.617 (0.291, 1.306)
Male	Baseline to T2D	1.031 (0.994, 1.070)	1.032 (0.984, 1.082)	1.066 (0.978, 1.162)	1.023 (0.974, 1.075)	1.077 (0.985, 1.177)
	T2D to MIC	0.984 (0.860, 1.126)	0.987 (0.888, 1.096)	0.979 (0.767, 1.250)	0.987 (0.856, 1.137)	0.973 (0.757, 1.252)
	T2D to DR	0.906 (0.765, 1.072)	0.943 (0.762, 1.167)	0.683 (0.438, 1.065)	0.999 (0.801, 1.247)	0.683 (0.434, 1.076)
	T2D to DN	0.866 (0.646, 1.159)	0.734 (0.509, 1.057)	1.277 (0.699, 2.332)	0.665 (0.447, 0.990)	1.085 (0.585, 2.014)
	T2D to DKD	1.077 (0.929, 1.248)	1.070 (0.883, 1.297)	1.188 (0.858, 1.644)	1.041 (0.849, 1.277)	1.209 (0.863, 1.694)
	MIC to Death	0.755 (0.548, 1.041)	0.864 (0.672, 1.112)	1.131 (0.658, 1.944)	0.705 (0.498, 0.998)	0.989 (0.568, 1.722)
	T2D to MAC	1.028 (0.919, 1.150)	0.985 (0.902, 1.074)	0.830 (0.668, 1.032)	1.065 (0.947, 1.196)	0.854 (0.682, 1.068)
	T2D to DCAD	0.975 (0.877, 1.083)	1.010 (0.883, 1.156)	0.834 (0.642, 1.084)	1.043 (0.906, 1.201)	0.850 (0.650, 1.113)
	T2D to DCVD	0.953 (0.783, 1.161)	0.994 (0.772, 1.280)	0.776 (0.473, 1.275)	1.038 (0.798, 1.351)	0.789 (0.474, 1.315)
	T2D to DPAD	0.898 (0.735, 1.097)	0.856 (0.665, 1.103)	0.934 (0.589, 1.481)	0.853 (0.652, 1.115)	0.874 (0.544, 1.402)
	MAC to Death	0.788 (0.620, 1.002)	0.847 (0.697, 1.030)	0.935 (0.598, 1.463)	0.780 (0.607, 1.003)	0.831 (0.524, 1.320)
BMI[‡]						
Normal or Underweight	Baseline to T2D	1.005 (0.917, 1.102)	1.007 (0.897, 1.131)	1.004 (0.806, 1.251)	1.006 (0.891, 1.136)	1.006 (0.803, 1.261)

	T2D to MIC	0.888 (0.683, 1.156)	0.890 (0.636, 1.245)	0.758 (0.393, 1.465)	0.926 (0.650, 1.320)	0.736 (0.376, 1.441)
	T2D to DR	0.968 (0.623, 1.504)	1.044 (0.596, 1.829)	0.687 (0.206, 2.285)	1.121 (0.625, 2.010)	0.737 (0.216, 2.512)
	T2D to DN	0.889 (0.433, 1.826)	0.772 (0.305, 1.953)	1.226 (0.262, 5.734)	0.725 (0.261, 2.011)	1.094 (0.225, 5.316)
	T2D to DKD	0.943 (0.653, 1.362)	0.939 (0.584, 1.511)	0.892 (0.378, 2.104)	0.946 (0.573, 1.562)	0.868 (0.357, 2.108)
	MIC to Death	0.798 (0.350, 1.819)	0.964 (0.335, 2.776)	0.264 (0.028, 2.522)	1.261 (0.413, 3.850)	0.282 (0.029, 2.767)
	T2D to MAC	0.913 (0.718, 1.159)	1.007 (0.738, 1.373)	0.563 (0.293, 1.081)	1.116 (0.809, 1.540)	0.590 (0.302, 1.151)
	T2D to DCAD	1.004 (0.752, 1.339)	1.234 (0.841, 1.811)	0.480 (0.206, 1.115)	1.422 (0.959, 2.108)	0.588 (0.250, 1.379)
	T2D to DCVD	0.777 (0.478, 1.264)	0.692 (0.371, 1.291)	0.839 (0.287, 2.450)	0.696 (0.356, 1.362)	0.729 (0.242, 2.195)
	T2D to DPAD	0.302 (0.143, 0.640)	0.288 (0.126, 0.658)	0.042 (0.001, 3.023)	0.401 (0.176, 0.914)	0.054 (0.002, 1.672)
	MAC to Death	0.799 (0.415, 1.541)	0.593 (0.287, 1.225)	6.676 (1.151, 38.737)	0.481 (0.227, 1.020)	5.320 (0.890, 31.812)
Overweight or Obese	Baseline to T2D	1.055 (1.024, 1.086)	1.055 (1.016, 1.096)	1.117 (1.043, 1.195)	1.040 (0.999, 1.082)	1.136 (1.059, 1.219)
	T2D to MIC	0.924 (0.851, 1.003)	0.906 (0.815, 1.006)	0.903 (0.745, 1.094)	0.914 (0.818, 1.021)	0.869 (0.713, 1.059)
	T2D to DR	0.879 (0.775, 0.998)	0.887 (0.756, 1.040)	0.727 (0.529, 0.999)	0.928 (0.786, 1.096)	0.704 (0.508, 0.975)
	T2D to DN	0.826 (0.644, 1.060)	0.750 (0.551, 1.023)	0.951 (0.539, 1.680)	0.733 (0.527, 1.019)	0.838 (0.468, 1.499)
	T2D to DKD	0.961 (0.855, 1.080)	0.923 (0.793, 1.073)	1.043 (0.805, 1.351)	0.905 (0.771, 1.063)	0.999 (0.765, 1.305)
	MIC to Death	1.009 (0.828, 1.230)	0.943 (0.732, 1.214)	1.263 (0.821, 1.943)	0.890 (0.678, 1.167)	1.206 (0.774, 1.878)
	T2D to MAC	0.981 (0.910, 1.059)	1.007 (0.913, 1.110)	0.882 (0.734, 1.060)	1.030 (0.930, 1.141)	0.894 (0.740, 1.080)
	T2D to DCAD	0.972 (0.887, 1.066)	0.978 (0.869, 1.099)	0.989 (0.875, 1.119)	1.043 (0.906, 1.201)	0.921 (0.737, 1.152)
	T2D to DCVD	0.959 (0.810, 1.134)	1.006 (0.811, 1.248)	1.054 (0.843, 1.319)	1.038 (0.798, 1.351)	0.785 (0.507, 1.216)
	T2D to DPAD	0.990 (0.825, 1.188)	0.961 (0.759, 1.217)	0.944 (0.735, 1.212)	0.853 (0.652, 1.115)	1.043 (0.685, 1.588)
	MAC to Death	0.886 (0.747, 1.052)	0.838 (0.677, 1.036)	0.959 (0.648, 1.419)	0.831 (0.665, 1.038)	0.876 (0.584, 1.316)

Abbreviations: T2D, type 2 diabetes; DR, diabetic retinopathy; DN, diabetic neuropathy; DKD, diabetic kidney disease; MIC, diabetic microvascular complications (including DR, DR or DKD); DCAD, diabetic coronary artery disease; DCVD, diabetic cerebrovascular disease; DPAD, diabetic peripheral vascular disease; MAC, diabetic macrovascular complications (including DCAD, DCVD, or DPAD).

*Multi-state model was adjusted for sex, Townsend deprivation index, income, education category, body mass index, smoking status, alcohol intake frequency, physical

activity, hypertension, healthy diet, sleep duration, sedentary time, genetic principal components of ancestry (first 10 columns).

†Multi-state model was adjusted for age, Townsend deprivation index, income, education category, body mass index, smoking status, alcohol intake frequency, physical activity, hypertension, healthy diet, sleep duration, sedentary time, genetic principal components of ancestry (first 10 columns).

‡Multi-state model was adjusted for age, sex, Townsend deprivation index, income, education category, smoking status, alcohol intake frequency, physical activity, hypertension, healthy diet, sleep duration, sedentary time, genetic principal components of ancestry (first 10 columns).

Table S7. Sensitivity analyses of the associations between rs10830963 and transitions from baseline to T2D, T2D comorbidities, and then death.

Transitions	Additive Model	Dominant Model	Recessive Model	Codominant Model (CG/GC)	Codominant Model (GG)
Excluding participants with cancer at baseline					
Baseline to T2D	1.053 (1.023, 1.085)	1.055 (1.016, 1.096)	1.109 (1.036, 1.186)	1.041 (1.001, 1.083)	1.128 (1.052, 1.210)
T2D to MIC	0.905 (0.833, 0.983)	0.887 (0.798, 0.986)	0.863 (0.710, 1.049)	0.901 (0.806, 1.007)	0.826 (0.676, 1.008)
T2D to DR	0.855 (0.754, 0.971)	0.857 (0.730, 1.006)	0.698 (0.506, 0.963)	0.901 (0.762, 1.065)	0.668 (0.480, 0.928)
T2D to DN	0.818 (0.638, 1.048)	0.748 (0.550, 1.016)	0.911 (0.514, 1.614)	0.737 (0.532, 1.020)	0.803 (0.448, 1.442)
T2D to DKD	0.971 (0.863, 1.093)	0.940 (0.806, 1.096)	1.039 (0.800, 1.350)	0.925 (0.785, 1.089)	1.005 (0.767, 1.316)
MIC to Death	0.938 (0.762, 1.155)	0.863 (0.663, 1.122)	1.162 (0.730, 1.847)	0.819 (0.617, 1.087)	1.077 (0.670, 1.732)
T2D to MAC	0.976 (0.905, 1.052)	1.003 (0.910, 1.105)	0.868 (0.724, 1.041)	1.029 (0.930, 1.140)	0.880 (0.730, 1.060)
T2D to DCAD	0.980 (0.895, 1.074)	0.999 (0.889, 1.124)	0.900 (0.725, 1.117)	1.019 (0.901, 1.152)	0.907 (0.726, 1.135)
T2D to DCVD	0.940 (0.798, 1.109)	0.963 (0.779, 1.190)	0.806 (0.539, 1.204)	0.999 (0.800, 1.246)	0.805 (0.533, 1.218)
T2D to DPAD	0.888 (0.740, 1.067)	0.848 (0.672, 1.071)	0.905 (0.594, 1.378)	0.849 (0.663, 1.087)	0.844 (0.548, 1.301)
MAC to Death	0.863 (0.728, 1.024)	0.822 (0.664, 1.017)	0.883 (0.597, 1.307)	0.825 (0.661, 1.030)	0.804 (0.535, 1.206)
Additional adjustment for the use of oral hypoglycemic drugs					
Baseline to T2D	1.056 (1.027, 1.086)	1.060 (1.022, 1.099)	1.109 (1.039, 1.183)	1.046 (1.007, 1.086)	1.131 (1.057, 1.209)
T2D to MIC	0.918 (0.849, 0.993)	0.901 (0.815, 0.996)	0.886 (0.737, 1.065)	0.912 (0.821, 1.014)	0.852 (0.705, 1.030)
T2D to DR	0.882 (0.782, 0.995)	0.896 (0.769, 1.043)	0.714 (0.526, 0.971)	0.940 (0.802, 1.103)	0.696 (0.508, 0.952)
T2D to DN	0.832 (0.657, 1.053)	0.751 (0.560, 1.006)	0.986 (0.580, 1.675)	0.728 (0.533, 0.994)	0.866 (0.503, 1.491)
T2D to DKD	0.959 (0.858, 1.072)	0.923 (0.800, 1.066)	1.028 (0.803, 1.316)	0.909 (0.780, 1.059)	0.987 (0.765, 1.274)
MIC to Death	0.988 (0.819, 1.192)	0.925 (0.727, 1.175)	1.211 (0.798, 1.837)	0.879 (0.679, 1.136)	1.152 (0.751, 1.766)
T2D to MAC	0.976 (0.908, 1.049)	1.006 (0.917, 1.104)	0.854 (0.716, 1.018)	1.036 (0.940, 1.141)	0.867 (0.723, 1.040)
T2D to DCAD	0.975 (0.894, 1.064)	0.995 (0.890, 1.113)	0.885 (0.718, 1.091)	1.017 (0.905, 1.143)	0.892 (0.719, 1.106)
T2D to DCVD	0.945 (0.807, 1.106)	0.976 (0.798, 1.195)	0.785 (0.530, 1.163)	1.017 (0.824, 1.256)	0.791 (0.528, 1.185)
T2D to DPAD	0.900 (0.756, 1.072)	0.861 (0.689, 1.076)	0.927 (0.619, 1.388)	0.859 (0.678, 1.087)	0.870 (0.575, 1.317)

MAC to Death	0.854 (0.727, 1.004)	0.802 (0.657, 0.978)	0.918 (0.633, 1.333)	0.797 (0.648, 0.982)	0.825 (0.562, 1.213)
Excluding T2D occurred in the first year of follow-up					
Baseline to T2D	1.043 (1.013, 1.073)	1.042 (1.005, 1.081)	1.094 (1.023, 1.169)	1.030 (0.991, 1.070)	1.107 (1.034, 1.186)
T2D to MIC	0.918 (0.848, 0.994)	0.902 (0.815, 0.999)	0.881 (0.730, 1.064)	0.914 (0.821, 1.017)	0.848 (0.699, 1.029)
T2D to DR	0.900 (0.797, 1.017)	0.917 (0.786, 1.070)	0.741 (0.545, 1.007)	0.959 (0.816, 1.127)	0.727 (0.531, 0.997)
T2D to DN	0.814 (0.640, 1.037)	0.732 (0.543, 0.986)	0.967 (0.559, 1.672)	0.710 (0.517, 0.976)	0.842 (0.481, 1.474)
T2D to DKD	0.945 (0.844, 1.059)	0.911 (0.787, 1.054)	0.999 (0.773, 1.290)	0.901 (0.771, 1.052)	0.955 (0.734, 1.244)
MIC to Death	0.992 (0.820, 1.200)	0.923 (0.723, 1.179)	1.235 (0.812, 1.878)	0.873 (0.671, 1.135)	1.171 (0.761, 1.801)
T2D to MAC	0.964 (0.895, 1.038)	0.989 (0.900, 1.087)	0.844 (0.703, 1.014)	1.018 (0.922, 1.124)	0.851 (0.705, 1.027)
T2D to DCAD	0.964 (0.881, 1.055)	0.980 (0.874, 1.099)	0.872 (0.701, 1.084)	1.003 (0.890, 1.130)	0.873 (0.697, 1.092)
T2D to DCVD	0.928 (0.790, 1.091)	0.956 (0.779, 1.173)	0.762 (0.507, 1.145)	0.998 (0.806, 1.236)	0.761 (0.501, 1.156)
T2D to DPAD	0.898 (0.751, 1.072)	0.845 (0.674, 1.059)	0.972 (0.649, 1.456)	0.833 (0.655, 1.059)	0.901 (0.595, 1.364)
MAC to Death	0.829 (0.700, 0.982)	0.764 (0.621, 0.940)	0.933 (0.633, 1.374)	0.755 (0.608, 0.938)	0.817 (0.547, 1.219)
Different time interval[§] (0.5-year)					
Baseline to T2D	1.048 (1.019, 1.078)	1.048 (1.011, 1.086)	1.034 (0.996, 1.074)	1.121 (1.048, 1.199)	1.098 (1.024, 1.178)
T2D to MIC	0.904 (0.835, 0.978)	0.882 (0.798, 0.975)	0.893 (0.803, 0.993)	0.832 (0.688, 1.007)	0.879 (0.721, 1.070)
T2D to DR	0.864 (0.766, 0.974)	0.871 (0.749, 1.013)	0.916 (0.782, 1.072)	0.671 (0.490, 0.918)	0.733 (0.531, 1.011)
T2D to DN	0.812 (0.641, 1.028)	0.724 (0.541, 0.970)	0.699 (0.512, 0.956)	0.849 (0.494, 1.460)	0.871 (0.497, 1.525)
T2D to DKD	0.963 (0.862, 1.076)	0.928 (0.803, 1.071)	0.912 (0.783, 1.063)	0.996 (0.771, 1.285)	1.012 (0.774, 1.324)
MIC to Death	0.992 (0.820, 1.201)	0.919 (0.721, 1.172)	0.865 (0.666, 1.123)	1.190 (0.776, 1.826)	1.188 (0.770, 1.831)
T2D to MAC	0.985 (0.916, 1.060)	1.014 (0.924, 1.113)	1.040 (0.943, 1.146)	0.895 (0.747, 1.072)	0.886 (0.731, 1.074)
T2D to DCAD	0.982 (0.900, 1.072)	0.997 (0.891, 1.115)	1.013 (0.900, 1.139)	0.923 (0.745, 1.143)	0.893 (0.708, 1.127)
T2D to DCVD	0.922 (0.787, 1.080)	0.950 (0.777, 1.162)	0.994 (0.806, 1.226)	0.748 (0.497, 1.128)	0.834 (0.548, 1.268)
T2D to DPAD	0.906 (0.758, 1.082)	0.868 (0.692, 1.089)	0.866 (0.681, 1.101)	0.878 (0.575, 1.339)	0.938 (0.619, 1.422)
MAC to Death	0.862 (0.731, 1.017)	0.802 (0.654, 0.984)	0.792 (0.639, 0.982)	0.856 (0.582, 1.259)	0.788 (0.521, 1.192)
Different time interval[§] (1-year)					

Baseline to T2D	1.047 (1.018, 1.077)	1.046 (1.009, 1.085)	1.105 (1.036, 1.179)	1.032 (0.994, 1.072)	1.120 (1.048, 1.198)
T2D to MIC	0.898 (0.830, 0.973)	0.877 (0.793, 0.970)	0.860 (0.712, 1.037)	0.891 (0.801, 0.990)	0.818 (0.675, 0.992)
T2D to DR	0.864 (0.766, 0.974)	0.871 (0.748, 1.013)	0.699 (0.514, 0.950)	0.915 (0.781, 1.071)	0.673 (0.492, 0.921)
T2D to DN	0.798 (0.629, 1.014)	0.703 (0.523, 0.943)	0.999 (0.588, 1.698)	0.673 (0.491, 0.923)	0.852 (0.496, 1.466)
T2D to DKD	0.961 (0.859, 1.074)	0.919 (0.796, 1.062)	1.051 (0.820, 1.345)	0.900 (0.772, 1.050)	1.005 (0.778, 1.297)
MIC to Death	0.984 (0.812, 1.193)	0.912 (0.714, 1.166)	1.238 (0.811, 1.891)	0.862 (0.662, 1.121)	1.168 (0.756, 1.804)
T2D to MAC	0.988 (0.918, 1.063)	1.021 (0.930, 1.123)	0.871 (0.729, 1.041)	1.050 (0.951, 1.158)	0.890 (0.741, 1.070)
T2D to DCAD	0.976 (0.894, 1.066)	0.997 (0.891, 1.116)	0.885 (0.717, 1.094)	1.019 (0.906, 1.146)	0.893 (0.718, 1.110)
T2D to DCVD	0.915 (0.780, 1.074)	0.935 (0.763, 1.145)	0.763 (0.511, 1.137)	0.974 (0.788, 1.204)	0.754 (0.500, 1.136)
T2D to DPAD	0.910 (0.762, 1.088)	0.876 (0.697, 1.099)	0.932 (0.618, 1.406)	0.874 (0.687, 1.113)	0.881 (0.577, 1.344)
MAC to Death	0.883 (0.745, 1.046)	0.832 (0.673, 1.027)	0.959 (0.650, 1.416)	0.824 (0.660, 1.028)	0.875 (0.585, 1.309)
Different time interval[§] (3-year)					
Baseline to T2D	1.051 (1.022, 1.081)	1.049 (1.012, 1.088)	1.116 (1.046, 1.192)	1.033 (0.995, 1.073)	1.133 (1.059, 1.211)
T2D to MIC	0.905 (0.833, 0.983)	0.867 (0.780, 0.962)	0.936 (0.773, 1.133)	0.864 (0.773, 0.965)	0.880 (0.723, 1.070)
T2D to DR	0.898 (0.795, 1.014)	0.907 (0.778, 1.058)	0.756 (0.556, 1.027)	0.944 (0.804, 1.108)	0.737 (0.539, 1.010)
T2D to DN	0.808 (0.636, 1.028)	0.709 (0.527, 0.955)	1.030 (0.606, 1.751)	0.675 (0.491, 0.929)	0.883 (0.513, 1.518)
T2D to DKD	0.995 (0.888, 1.114)	0.958 (0.827, 1.110)	1.105 (0.861, 1.418)	0.933 (0.797, 1.091)	1.073 (0.829, 1.389)
MIC to Death	1.002 (0.812, 1.237)	0.901 (0.688, 1.180)	1.391 (0.893, 2.168)	0.825 (0.616, 1.104)	1.289 (0.817, 2.036)
T2D to MAC	0.969 (0.897, 1.048)	0.993 (0.899, 1.096)	0.861 (0.711, 1.041)	1.019 (0.918, 1.130)	0.868 (0.713, 1.056)
T2D to DCAD	0.974 (0.891, 1.065)	0.997 (0.890, 1.117)	0.870 (0.700, 1.082)	1.022 (0.907, 1.151)	0.878 (0.702, 1.099)
T2D to DCVD	0.951 (0.811, 1.116)	0.974 (0.794, 1.194)	0.826 (0.558, 1.224)	1.005 (0.812, 1.244)	0.828 (0.553, 1.240)
T2D to DPAD	0.931 (0.775, 1.117)	0.906 (0.718, 1.144)	0.937 (0.611, 1.439)	0.908 (0.710, 1.160)	0.900 (0.579, 1.397)
MAC to Death	0.872 (0.723, 1.053)	0.807 (0.639, 1.019)	1.001 (0.653, 1.533)	0.790 (0.618, 1.010)	0.899 (0.580, 1.396)
Different time interval[§] (5-year)					
Baseline to T2D	1.043 (1.014, 1.074)	1.039 (1.002, 1.077)	1.111 (1.040, 1.187)	1.023 (0.984, 1.063)	1.122 (1.048, 1.201)
T2D to MIC	0.891 (0.817, 0.972)	0.852 (0.764, 0.951)	0.911 (0.743, 1.116)	0.853 (0.759, 0.958)	0.852 (0.692, 1.049)

T2D to DR	0.881 (0.778, 0.998)	0.888 (0.760, 1.038)	0.728 (0.528, 1.004)	0.926 (0.787, 1.089)	0.705 (0.507, 0.979)
T2D to DN	0.790 (0.617, 1.012)	0.689 (0.509, 0.933)	1.027 (0.593, 1.778)	0.655 (0.473, 0.907)	0.871 (0.498, 1.525)
T2D to DKD	1.004 (0.895, 1.127)	0.964 (0.831, 1.118)	1.142 (0.888, 1.469)	0.933 (0.796, 1.093)	1.109 (0.855, 1.438)
MIC to Death	0.995 (0.792, 1.251)	0.898 (0.671, 1.200)	1.374 (0.844, 2.235)	0.827 (0.605, 1.131)	1.273 (0.771, 2.102)
T2D to MAC	1.006 (0.923, 1.096)	1.034 (0.927, 1.155)	0.919 (0.747, 1.130)	1.053 (0.939, 1.182)	0.941 (0.760, 1.165)
T2D to DCAD	0.981 (0.895, 1.076)	0.991 (0.882, 1.115)	0.927 (0.742, 1.157)	1.004 (0.888, 1.135)	0.928 (0.739, 1.167)
T2D to DCVD	0.957 (0.812, 1.127)	0.981 (0.796, 1.209)	0.828 (0.551, 1.245)	1.012 (0.814, 1.259)	0.832 (0.547, 1.266)
T2D to DPAD	0.925 (0.767, 1.116)	0.921 (0.727, 1.167)	0.856 (0.542, 1.353)	0.939 (0.733, 1.204)	0.834 (0.521, 1.333)
MAC to Death	0.911 (0.732, 1.134)	0.885 (0.674, 1.162)	0.918 (0.546, 1.544)	0.888 (0.668, 1.180)	0.868 (0.508, 1.484)
Excluding participants who entered different states on the same date					
Baseline to T2D	1.053 (1.022, 1.085)	1.055 (1.015, 1.096)	1.109 (1.035, 1.189)	1.041 (0.999, 1.084)	1.128 (1.050, 1.212)
T2D to MIC	0.901 (0.815, 0.996)	0.879 (0.774, 0.999)	0.865 (0.683, 1.096)	0.892 (0.779, 1.021)	0.824 (0.646, 1.051)
T2D to DR	0.855 (0.702, 1.040)	0.832 (0.711, 0.974)	0.584 (0.380, 0.899)	0.919 (0.750, 1.126)	0.563 (0.363, 0.874)
T2D to DN	0.652 (0.450, 0.944)	0.747 (0.552, 1.011)	0.910 (0.460, 1.798)	0.630 (0.424, 0.938)	0.759 (0.379, 1.521)
T2D to DKD	0.964 (0.802, 1.158)	1.005 (0.874, 1.157)	1.139 (0.843, 1.539)	0.931 (0.764, 1.133)	1.105 (0.809, 1.509)
MIC to Death	1.105 (0.861, 1.418)	1.027 (0.748, 1.411)	1.553 (0.905, 2.663)	0.942 (0.670, 1.325)	1.516 (0.869, 2.644)
T2D to MAC	0.954 (0.863, 1.054)	0.979 (0.862, 1.112)	0.822 (0.643, 1.052)	1.012 (0.886, 1.156)	0.827 (0.642, 1.064)
T2D to DCAD	1.014 (0.867, 1.186)	1.000 (0.885, 1.129)	0.954 (0.718, 1.269)	1.024 (0.869, 1.207)	0.964 (0.719, 1.295)
T2D to DCVD	0.872 (0.669, 1.137)	0.874 (0.708, 1.078)	0.744 (0.440, 1.259)	0.907 (0.688, 1.197)	0.714 (0.417, 1.223)
T2D to DPAD	0.806 (0.598, 1.087)	0.798 (0.627, 1.016)	0.563 (0.288, 1.104)	0.866 (0.636, 1.181)	0.530 (0.268, 1.051)
MAC to Death	0.843 (0.661, 1.073)	0.776 (0.574, 1.048)	0.943 (0.547, 1.625)	0.765 (0.557, 1.050)	0.832 (0.474, 1.461)

Abbreviations: T2D, type 2 diabetes; DR, diabetic retinopathy; DN, diabetic neuropathy; DKD, diabetic kidney disease; MIC, diabetic microvascular complications (including DR, DR or DKD); DCAD, diabetic coronary artery disease; DCVD, diabetic cerebrovascular disease; DPAD, diabetic peripheral vascular disease; MAC, diabetic macrovascular complications (including DCAD, DCVD, or DPAD).

[§]For participants who entered different states on the same day, we calculated the entering date of the prior state using additional four different time intervals, i.e., 0.5-year, 1-year, 3-year, and 5-year.

Multi-state model was adjusted for sex, age, Townsend deprivation index, income, education category, body mass index, smoking status, alcohol intake frequency, physical activity, hypertension, healthy diet, sleep duration, sedentary time, genetic principal components of ancestry (first 10 columns).

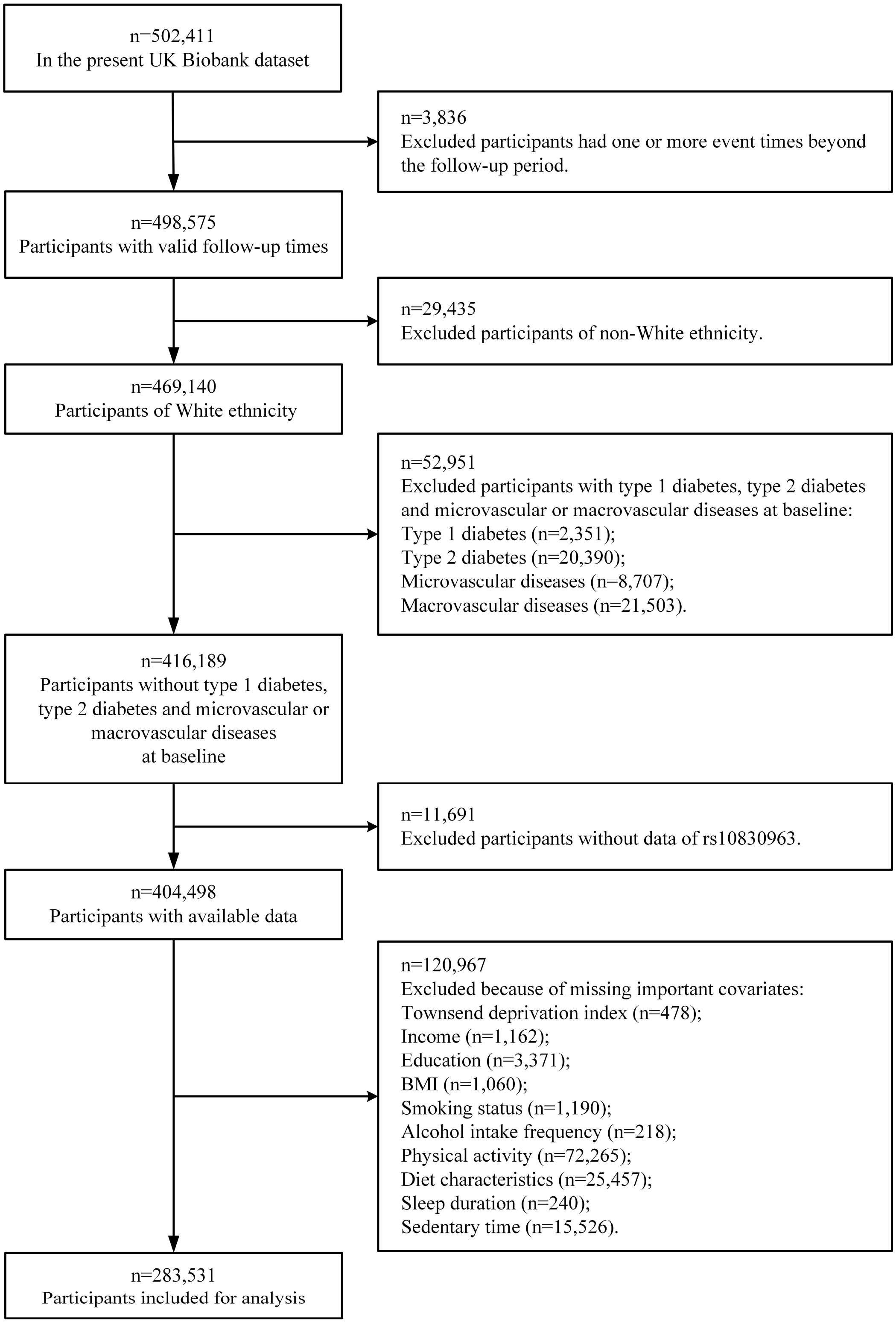

Figure S1. The flow chart for the inclusion and exclusion of study participants.
